# Role of Temperature in the Growth of Silver Nanoparticles Through a Synergetic Reduction Approach

**DOI:** 10.1007/s11671-010-9780-1

**Published:** 2010-09-23

**Authors:** XC Jiang, WM Chen, CY Chen, SX Xiong, AB Yu

**Affiliations:** 1 School of Materials Science and Engineering, University of New South Wales, Sydney, NSW 2052, Australia; 2 Key Laboratory for Anisotropy and Texture of Materials (Ministry of Education), School of Materials and Metallurgy, Northeastern University, Shenyang 110004, China

**Keywords:** Silver nanoparticles, Nanoplates, Reaction temperature, Thermodynamics effect

## Abstract

This study presents the role of reaction temperature in the formation and growth of silver nanoparticles through a synergetic reduction approach using two or three reducing agents simultaneously. By this approach, the shape-/size-controlled silver nanoparticles (plates and spheres) can be generated under mild conditions. It was found that the reaction temperature could play a key role in particle growth and shape/size control, especially for silver nanoplates. These nanoplates could exhibit an intensive surface plasmon resonance in the wavelength range of 700–1,400 nm in the UV–vis spectrum depending upon their shapes and sizes, which make them useful for optical applications, such as optical probes, ionic sensing, and biochemical sensors. A detailed analysis conducted in this study clearly shows that the reaction temperature can greatly influence reaction rate, and hence the particle characteristics. The findings would be useful for optimization of experimental parameters for shape-controlled synthesis of other metallic nanoparticles (e.g., Au, Cu, Pt, and Pd) with desirable functional properties.

## Introduction

Precious metallic nanoparticles have become more attractive because of their fascinating functional properties such as optical, electronic, and physicochemical properties due to their high surface-to-volume effect [1-7]. In most cases, the properties are heavily affected by the morphology, size, and size distribution of nanoparticles. The shape/size control of metal nanocrystals is critical and has increasingly attracted attention in the past [8-27]. Of the achieved nanoparticles such far, low-dimensional (LD) silver nanostructures (e.g., plates, discs, rods, and wires) have been extensively investigated because of an extreme degree of anisotropic geometry together with corners and/or edges (or ends) for generating maximum electromagnetic field enhancement. These have inspired not only the development of synthesis, growth, and mechanistic understanding but also the exploration of functional applications in many areas, such as near-field optical probes, optical sensors, surface-enhanced Raman spectroscopy (SERS), and biomedical labelling [8-27].

Many efforts have been made to control the formation and growth of silver nanoparticles with two-dimensional (2D) morphologies for unique functional properties and potential applications through chemical methods, such as photoinduced method [28-31], electrochemical method [32], ultrasonic-assistant method [33], solvothermal method [34-38], and templating method (e.g., 'soft' reverse micelles and 'hard' polystyrene spheres) [39-43]. Many methods have shown that the controlled growth of silver nanocrystals in solution requires specific reaction conditions (e.g., photo illumination with specially selected light wavelength or laser, and γ-ray radiation with a unique Co_60_ source). Among the experimental parameters, reaction temperature has been considered of great importance [34-55]. A variety of studies investigated the synthesis process at a fixed temperature, although the optimization process is probably performed. Unfortunately, little literature systematically studied the effect of the reaction temperature on the formation of silver nanoplates. For instance, Mirkin and co-workers [28,29] demonstrated a photochemical synthesis method for generating silver nanoprisms at room temperature. By extending this technique, both Maillard [30] and Callegari groups [31] have reported the formation of Ag nanoprisms through photoinduced conversion in which the shape and size can be directly influenced by illumination wavelengths at room temperature, while the formation of silver nanoprisms usually took a long time (a few days).

Other methods in literature also paid less attention to systematically discuss the function of the reaction temperature, although a certain temperature was applied. Henglein and Giersig [44] supposed a γ-irradiation method to synthesize colloidal silver sols at room temperature. Electrochemical reaction for the synthesis of anisotropic gold or silver nanoparticles in an aqueous electrolyte solution could happen at room temperature or higher temperatures [45,46]. For example, the surface condition and composition of the nanowires varied with electro-deposition time at 60°C under an applied potential of 1.6 V [47]. By applying a cathodic voltage to the microelectrode, silver nanowires could be deposited at ~150°C [48]. In addition, Chen and Carroll reported a surfactant-assisted method to generate silver nanodiscs (e.g., triangle, truncated, and spherical particles) in water by ageing at 40°C for a few hours [42]. Zhang et al. [38] used a water/PVP/*n*-pentanol ternary system to synthesize silver nanoprisms by heating at 95°C for 48 h, followed by obtaining a mixture of platelets with different morphologies/sizes. The polyol-mediated synthesis, as a solvothermal reduction method, has been widely studied by Xia and his colleagues who demonstrated the shape control of silver nanoplates through heating ethylene glycol at a relatively high temperature (e.g., 160°C), which could lead to the kinetics or thermodynamics growth depending on experimental parameters [32,34-36].

Recently, a synergetic reducing approach has been developed by our laboratory for the synthesis of silver nanoplates using two or three reducing agents including citric acid, L-ascorbic acid, and NaBH_4_ at room temperature [49-55]. The role of various components, such as reducing agents (citric acid, L-ascorbic acid, and NaBH_4_), pH, and concentration, has been investigated. Moreover, the stability, electrochemical property, and sensing application of silver plates in aqueous system have also been discussed in detail. However, the role of reaction temperature in the growth rate of silver nanoparticles is still not properly understood. This would impede the progress in the exploitation of shape-controlled synthesis and functional applications of silver nanoplates. A systematical study of the thermodynamics (temperature) effect on particle formation and growth is needed.

In this study, we investigate the role of the reaction temperature in the shape-controlled synthesis of silver nanoplates through a synergetic reduction approach. This is a part of our series studies in this area. The function of temperature in the formation and growth of silver nanoparticles will be identified by transmission electron microscopy (TEM) and UV–vis spectrometer techniques. The possible mechanisms on particle growth are finally discussed.

## Experimental Section

### Chemicals

Silver nitrate (AgNO_3, 9_9.9%), citric acid (99%), sodium citrate (99%), L-ascorbic acid (≥99.0%), sodium borohydride (NaBH_4_, 99%), and sodium bis(2-ethylhexyl) sulfosuccinate (NaAOT, 99%) are all purchased from Sigma–Aldrich and used as received without further treatment. All the solutions were freshly made for the synthesis of silver nanoparticles, especially the freshly made NaBH_4_ aqueous solution that was ice bathed before use. Ultra-pure water was used in all the synthesis processes.

### Synthesis of Anisotropic Silver Nanoparticles

The experimental procedure was performed according to our recent work with some modifications [49-55]. In a typical procedure, three steps were involved as followings. Firstly, 1.25 mL aqueous AgNO_3_ (0.02 M) and 2.5 mL NaAOT (0.02 M) solution were added into a 200-mL conical flask with ultra-pure water and then stirred for 10 min to ensure homogeneity. The final volume of mixture was then fixed at 100 mL, and the concentrations of AgNO_3_ and NaAOT were adjusted at 2.5 × 10^-4^ M and 5.0 × 10^-4^ M, respectively. Secondly, 1.20 mL citric acid (1.0 M) (citric acid/Ag^+^ = 40) and 0.30 mL L-ascorbic acid (Laa, 0.10 M) (Laa/Ag^+^ = 1.2) aqueous solution was added to the 100 mL solution and then stirred vigorously to obtain a homogeneous solution. The molar ratio of citric acid to silver ions was selected as high to 40 based on our recent study on the role of citric acid. At lower ratios of citric acid to silver (<12), the reaction will be very fast especially at a temperature over 30°C, where the data for TEM analysis and the UV–vis spectra are difficult to collect and record. Finally, 0.02 mL NaBH_4_ (0.002 M) aqueous solution was rapidly added into the above mixed solution and stirred for ~30 s. The colour of the reaction solution changed gradually from light yellow to purple, then pink, green, and finally blue.

The effect of the reaction temperature was systematically investigated by adjusting from 0 to 55°C. A higher temperature over 60°C was not considered in this work because it could result in reaction too quick to track using UV–vis and TEM techniques. For example, in a boiling solution, citric acid will become more active in reducing silver ions than a lower temperature (e.g., <60°C) [53,56,57], and the reaction can finish within a minute as observed. Other experimental parameters, such as the molar ratio of silver to other reactants, concentration of silver ions, L-ascorbic acid, and sodium borohydride, remain constant. To minimize the volume effect of the reactants, the total volume of the proposed system was basically kept at 100 mL by adjusting the concentration of reducing agent.

### Equipment

Transmission electron microscope (TEM) patterns were conducted under JEOM 1400 and operated at 100 kV. The specimen was prepared by dropping the solution onto the copper grids covered with amorphous carbon and air dried naturally. UV–vis absorption spectrum was obtained on a CARY 5G UV–visible Spectrophotometer (Varian) with a 1-cm quartz cell.

## Results and Discussion

**Figure 1:**
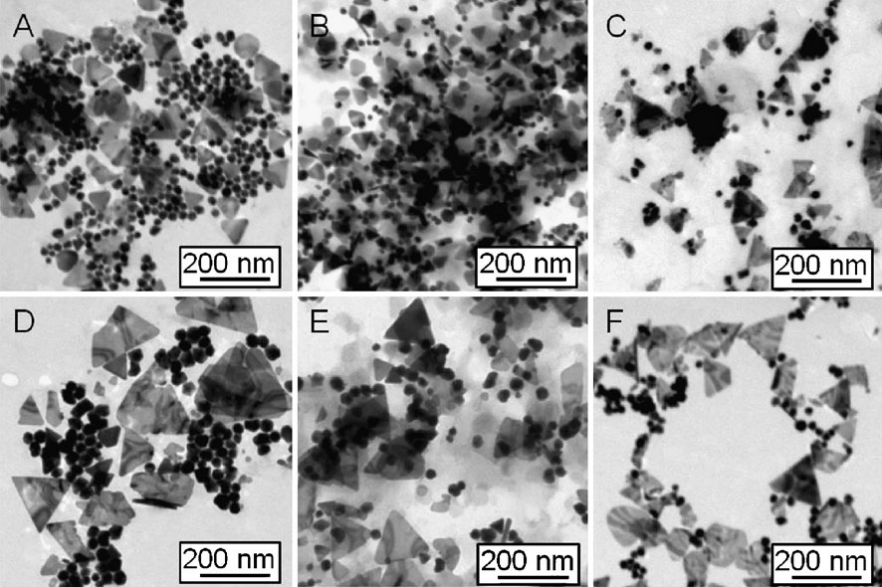
**TEM images of silver nanoparticles (*triangles plus spheres*) obtained in the reaction end at different temperatures: a 17; b 23; c 28; d 32; e 43; and f 55°C**.

Temperature is one of the key influence factors in chemical reactions. In order to investigate the influence of heating or cooling process in the synthesis of silver nanoplates, the solution temperature was cooled by ice-bath to ~0°C or heated from 17 to 23, 28, 32, 38, 43, 50, and 55°C, respectively. Other parameters were kept constant. The change of pH is estimated to be ΔpH ≤ 0.3 in the solution (pH ~ 5) when adjusting the molar ratio of [cit]/[Ag^+^] to 40 [53]. Figure 1 shows TEM images that silver nanoparticles can be formed in the proposed system at different temperatures, unfortunately, the triangular and spherical particles co-exist (Figure 1a–f).

Two possible reasons can be used for understanding the phenomena: first, at the initial stage, two or more kinds of nuclei or silver clusters with different shapes (e.g., plates, spheres, hexagonal, tetragonal, or octagonal) co-exist in the reaction system, which may lead to the formation of plate-like particles and/or spherical particles. The ratio of different shapes is dependent on the amount of the silver clusters with different geometry at the initial stage. Such a description has been reported in different synthesis method, such as ultrasonic-assistant method [33], solvothermal method [34-38], templating method (e.g., 'soft' reverse micelles and 'hard' polystyrene spheres) [39-43], and hydrochemical method [49-55]. The assumption can be proved by existence of $\text{A}{\text{g}}_{2}^{+}$ and $\text{A}{\text{g}}_{2}^{+}$(citrate) clusters identified by electrospray ionization mass spectrometry (ESI–MS) measurements, as demonstrated in our recent work [53]. The existence of other kinds of silver clusters (e.g., $\text{A}{\text{g}}_{4}^{2+}$, $\text{A}{\text{g}}_{8}^{4+}$, $\text{A}{\text{g}}_{3}^{+}$ or Ag_3_) has been investigated in the past. Henglein et al. [58] has shed some light on the nucleation process by controlling the generation of zero-valent atoms and thus their agglomeration into small clusters in a gamma-radiation-based synthesis. Silver clusters consisting of a few atoms, which do not possess metallic properties. The investigators found that silver ions were radiolytically reduced in AgClO_4_ solutions containing sodium poly(phosphate). In the initial stages of reduction, small clusters of silver are formed as well as colloidal particles of silver metal. In the early stages of the silver reduction, the clusters are the main products (e.g., $\text{A}{\text{g}}_{4}^{2+}$, $\text{A}{\text{g}}_{8}^{4+}$, $\text{A}{\text{g}}_{3}^{+}$ or Ag_3_). The clusters survive for a short time (~1 h). They absorb at 275, 300, and 325 nm, while the metallic particles absorb at 380 nm even longer.

Both UV/Vis spectroscopic and scanning tunnelling microscopic studies of these clusters suggested that $\text{A}{\text{g}}_{4}^{2+}$ and $\text{A}{\text{g}}_{8}^{4+}$ were the most abundant species involved in the nucleation stage [59]. Growth of these clusters into nanocrystals likely occurred through a combination of aggregation and atomic addition. Xia et al. [60] demonstrated that there exists a smaller cluster, $\text{A}{\text{g}}_{3}^{+}$ or Ag_3_, in the nucleation stage of a solution-phase synthesis that employs AgNO_3_ as a precursor to silver. These trimeric clusters can serve as nuclei for the addition of newly formed silver atoms and eventually lead to the formation of triangular nanoplates, while the $\text{A}{\text{g}}_{2}^{+}$, $\text{A}{\text{g}}_{4}^{2+}$, and $\text{A}{\text{g}}_{8}^{4+}$ might benefit for the formation of spheres. These authors also demonstrated that mass spectrometry provides a tool for simple identification and characterization of silver clusters possibly contained in aqueous AgNO_3_ solution. Since a mass spectrometer can separate and detect ions of different masses, it allows the different isotopes of a given element to be easily distinguished. The positively charged $\text{A}{\text{g}}_{3}^{+}$ cluster is a trimer whose ground state has an equilateral-triangular structure (^1^A_1_) and *D*_*3h*_ symmetry. The linear 1Σ_g_ state is predicted to lie approximately 1 eV above the ^1^A_1_ state. Thus, the $\text{A}{\text{g}}_{3}^{+}$ cluster should exist as a triangle, in the lowest-energy state. Similarly, the ground state of the neutral Ag_3_ cluster is a ^2^E' state with an equilateral-triangular structure (*D*_*3h*_ symmetry) [61]. The triangular configuration of the trimeric clusters may naturally result in a triangular shape for the nuclei and thus for the final products.

Second, the co-existence of different morphologies may be caused by the essential crystalline silver structure, face-centre-cubic (fcc) structure, which usually shows single-, twin-, or multiple-plane structures. This could play an important role in the formation of nanoparticles with different geometries. The ratios of plates or rods to spheres/near-spheres could be mainly determined by the amount of single or twin structures formed at the initial nucleation stage. Some approaches using surface control like surfactants or polymers molecules or assisted by templates will benefit for the formation and growth of non-spheres [28-31,34-43,49-55]. After a carefully comparison, with temperature increasing, the spheres seem to reduce in quantity. For example, at 17°C, the ratio of plates to spheres is around 1:1, while at 55°C, it reduces to 1:3. This indicates that the yield of silver nanoplates could be tuned by changing temperature under the reported conditions.

**Figure 2:**
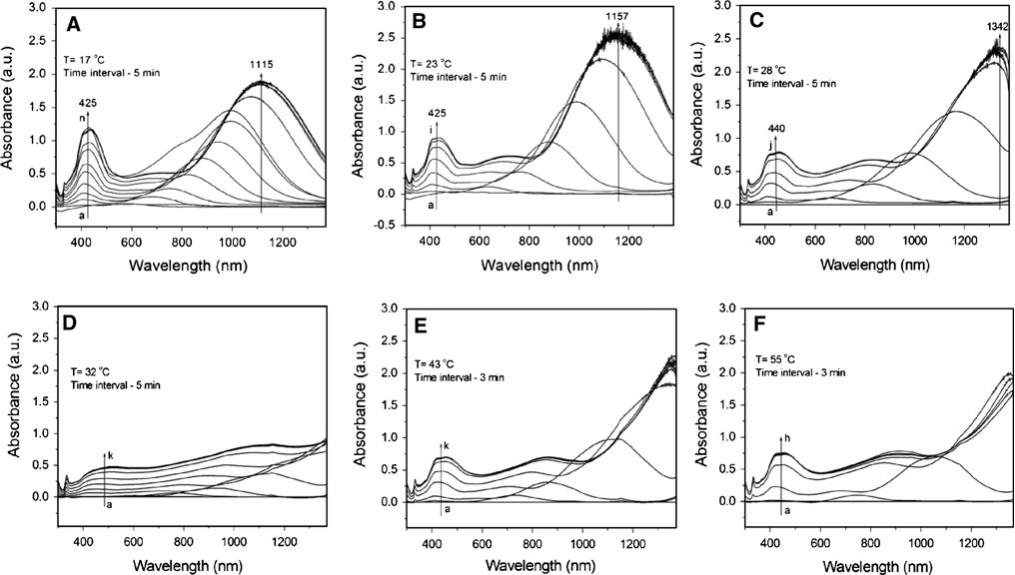
**UV–vis spectra of silver nanoparticles for the growth process obtained at different temperatures: a 17; b 23; (c) 28; d 32; e 43; and f 55°C**.

The formation and growth of the silver nanostructures was further identified by UV–vis spectroscopy. Surface plasmon resonances are typically studied as physical properties of metal nanostructures rather than chemical tools that can provide control over growth and ultimate particle dimensions. Figure 2 shows the UV–vis spectra recorded at different times, corresponding to the different temperatures of 17–55°C (a–f). In general, with temperature increasing, the strongest absorption band gradually shift to a longer wavelength, e.g., from λ = 1,115 nm at 17°C (Figure 2a) to λ = 1,157 nm at 23°C (Figure 2b) and to λ = 1,342 nm at 28°C (Figure 2c). While it is heated over 32°C, the strongest absorption band is beyond 1400 nm. This means that the size of silver nanoplates increases with temperature, which is in a good agreement with the TEM observations shown in Figure 1. In addition, the profile and growth trend of UV–vis spectra of silver particles at 32°C are apparently different from those happened at other temperatures (e.g., 17, 23, 28, 43, and 55°C). In the range of 350–600 nm, only a very weak absorption emerges centred at around 425 nm, while the strongest absorption band is beyond the detection wavelength range (300–1,400 nm). This suggests that the silver nanoplates are the main product, and the plates could be larger than those obtained at other temperatures (e.g., 17, 23, and 28°C). This is further confirmed by TEM observations (Figure 1). Moreover, the crystal structure and composition of silver nanoparticles has been investigated by high-resolution TEM (HRTEM), electron diffraction, and X-ray diffraction techniques (XRD) (the images/patterns are not shown here). The nanoplates are of single crystal structure, similar as those reported in our recent work [49-55].

**Figure 3:**
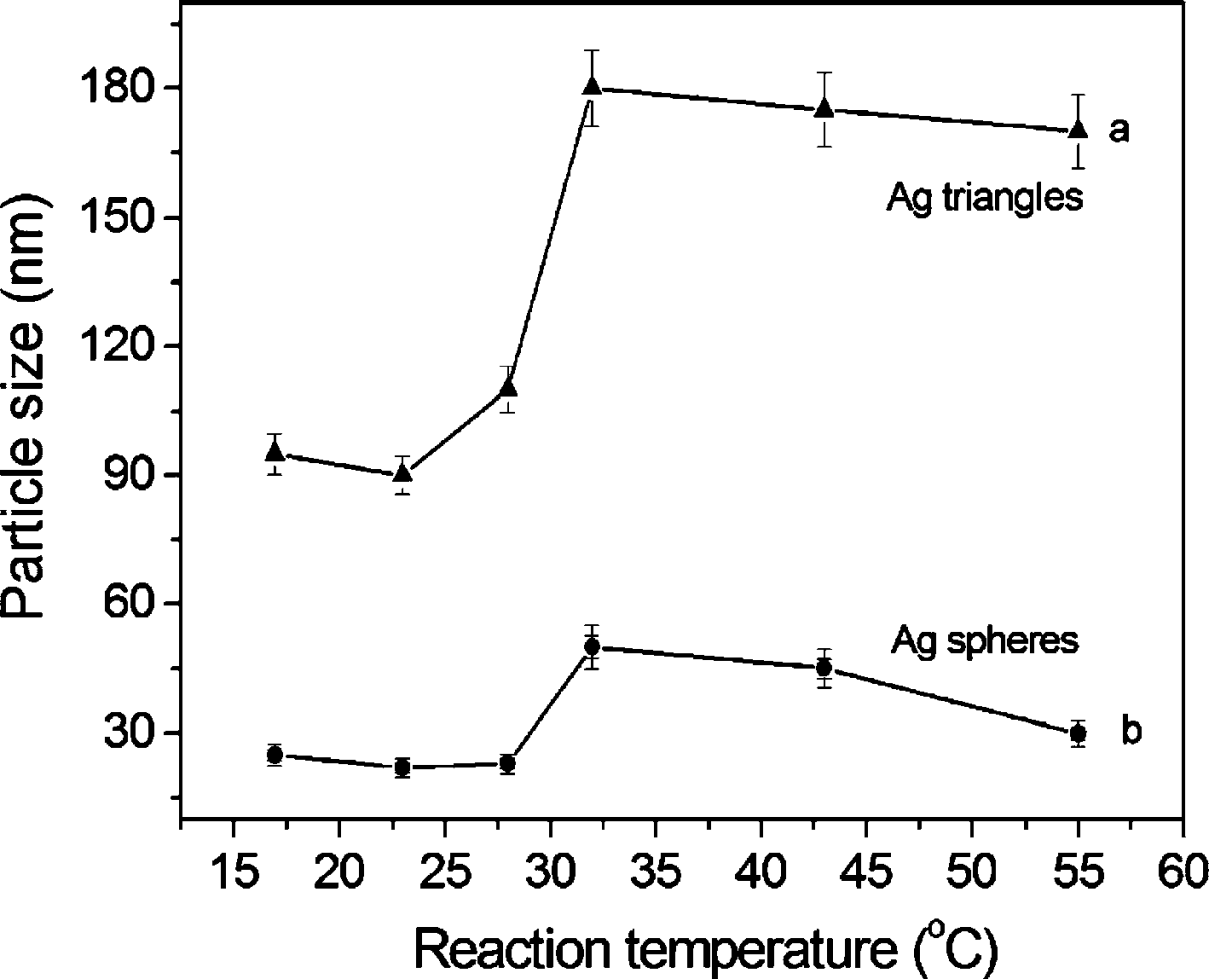
**The average size of silver nanoplates (*curve a*) and nanospheres (*curve b*) obtained at different temperatures from 17 to 55°C**.

To compare the particle size and growth trend, Figure 3 shows the average size of silver plates (curve a) and spheres (curve b) obtained at different temperatures from 17 to 55°C. With temperature increasing, the triangular silver nanoplates grow larger from 90 nm (17–28°C) to 180 nm (>32°C), according to the curve a (Figure 3). There is a significant jump in plate size growth. This is also confirmed by TEM image shown in Figure 1. This phenomenon is consistent with previous study on the bimodal growth of silver nanoprisms reported by Mirkin et al. [28,29]. The authors demonstrated that the observed bimodal growth process occurs through an edge-selective particle fusion mechanism, with four 'small' nanoprisms coming together in step-wise fusion to form a 'big' one. The fusion mechanism was confirmed by a few experimental observations in the photoinduced approach: (1) the cumulative edge length of triangular nanoprism increases nearly twice; (2) edge-selective growth occurs with no apparent change in nanostructure thickness; (3) detailed time-dependent UV–vis–NIR measurements show that the onset of the growth of the band at 1,065 nm (assigned to 'big') is significantly delayed in comparison with the growth of the band at 640 nm (assigned to 'small'). This indicates that the fusion of nanoprisms occurs only after 'small' nanoprisms have accumulated; and (4) a small population of dimer 2 and trimer 3 intermediates is observed during the early stages of 'small' particle growth. This means that the similar fusion growth of triangular particles could occur under an appropriate heating process (30–35°C). A detailed mechanism for these types of conversions remains to be determined.

Similar scenario was observed for the growth of spheres, and the size also shows a growth jump, i.e., the average size increases from 25 to 48 nm when temperature was raised from 17 to 32°C. While it is over 32°C, the average size of spheres decreases to ~45 nm (43°C) and ~30 nm (55°C), respectively. The jumped size for particles is probably caused by a fusion growth process. Tang et al. [62] reported the formation of CdTe nanorods/wires fused by spherical nanoparticles after the removal of surface ligands. Similar examples involving spherical particle fusion have also been reported by Penn et al. [63] who presented a mechanism for dislocation formation that may operate during early growth that involves attachment between two or more nanoparticles.

**Figure 4:**
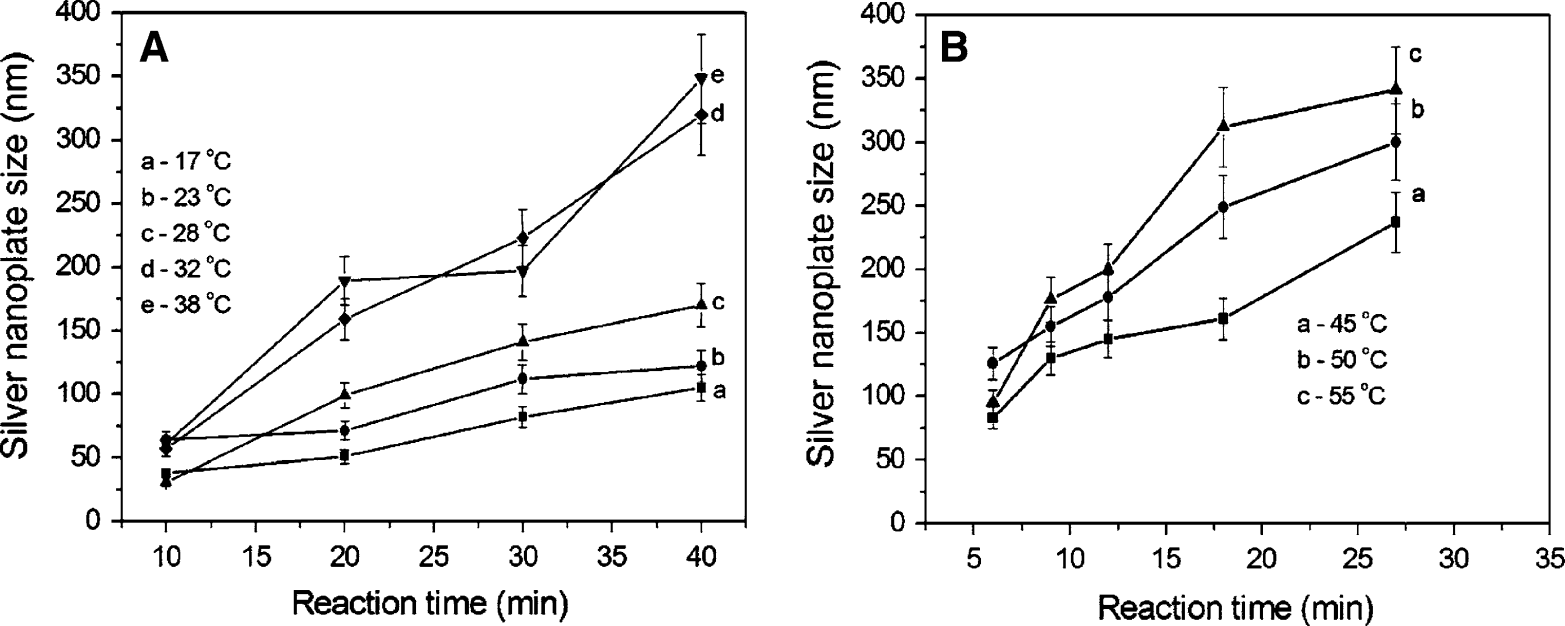
**The size evolution process of silver nanoparticles at different temperatures: a 17–38°C and b 45–55°C**.

To track the formation and growth with time, the dimensions of plates were checked. Figure 4 shows the growth process of silver plates at different temperature stages of 17–38°C (a) and 45–55°C (b). Because the data were collected at different time intervals of 5 min for lower temperatures (<38°C) and 3 min for relatively high temperatures (>42°C), the data were analysed separately. In general, the particle size increases with time at 17–55°C: from 90 to 180 nm for the edge length of triangular plates and from 25 to 48 nm for the diameter of silver spheres. The reaction rate denoted by the slope of particle growth curves shows that the higher the temperature the faster the particle grows. Additionally, it appears that a size jump occurred from 90 to 180 nm for plates and/or 25 to 48 nm for spheres when the temperature is around 32–38°C, as observed in TEM images (Figure 1d) and the growth trend (Figure 3). The possible fusion growth mechanism occurred at this stage has been discussed above. This is different from the observation of particle grow with time through Ostwald ripening process at room temperature [53]. The details in this growth mechanism need to be investigated in the near future. Nonetheless, the effect of temperature on particle size increase is significant under the reported conditions.

**Figure 5:**
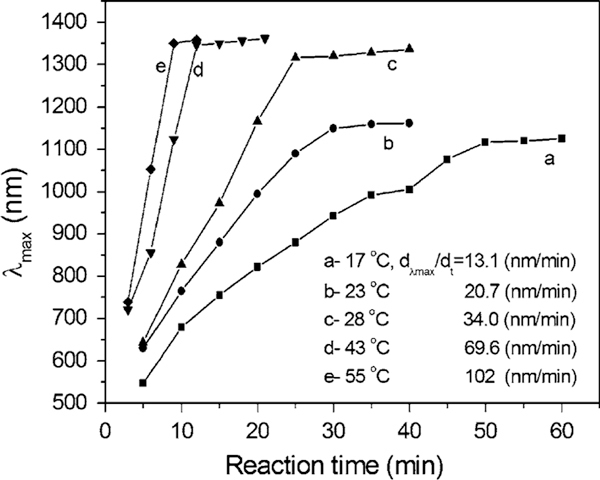
**The growth rate of silver nanoplates reflected by the relationship between λ_max_ and reaction time at different temperatures: a 17; b 23; c 28; d 43; and e 55°C**.

The effect of temperature can be further confirmed by the kinetics analysis. The relationship between the wavelength of the strongest plasmon resonance absorption (λ_max_) and reaction time (*t*) was plotted and shown in Figure 5. As well known, the position of the maximum absorption peak, λ_max_, is strongly dependent on the dimensions of the silver particles [27-43]. The value of dλ_max_/d*t* in this experiment reflects the reaction rate, estimated to be 13.1, 20.7, 34, 69.6, and 102, corresponding to the reaction temperatures of 17, 23, 28, 43, and 55°C, respectively. That is, the higher the reaction temperature the faster the silver nanoparticles grow. The value of dλ_max_/d*t* approaches zero when the reaction ends, at that moment the UV–vis spectrum does not change again. Meanwhile, the shift in the strongest plasmon resonance absorption to longer wavelengths is significant from 1,115 (17°C) to 1,157 (23°C), 1,342 (28°C), and beyond 1,400 nm (both 42 and 55°C) (Figure 2). This suggests that the particle size gradually increases, as also evidenced by TEM observations (Figure 1). The detailed kinetics study on the relationship between particle dimensions and plasmon resonance absorption position/intensity is under progress.

**Figure 6:**
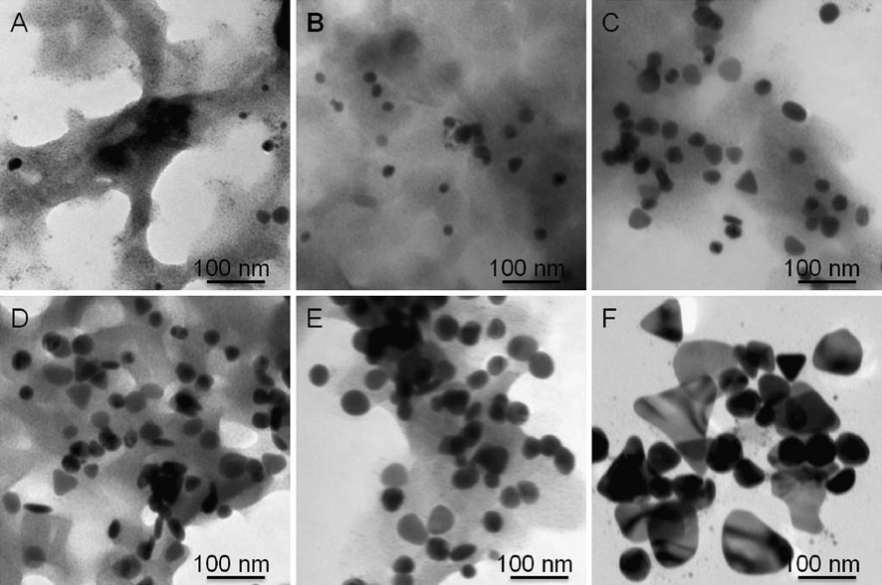
**TEM images showing the growth process of silver nanoparticles obtained at the temperature of ~0°C**.

In comparison, the effect of a low temperature (~0°C) on the formation and growth of silver nanoparticles was also investigated. Figure 6 shows the formation and growth process of silver nanoparticles. It is clear that some small clusters with a few nanometres were formed at the initial stage (a). After ~ 30 min, the clusters grow larger into spherical particles with a diameter of ~20 nm (b). A few triangular plates with edge length of ~50 nm emerge in the product after 1 h (c), and the spherical particles (40–60 nm in diameter) are the main form. After ~3 h, big triangular plates with edge lengths of ~80 nm were formed, along with some large spherical particles with the diameter of ~60 nm (F). Unfortunately, the shape and size of the particles obtained at ~0°C is not uniform.

**Figure 7:**
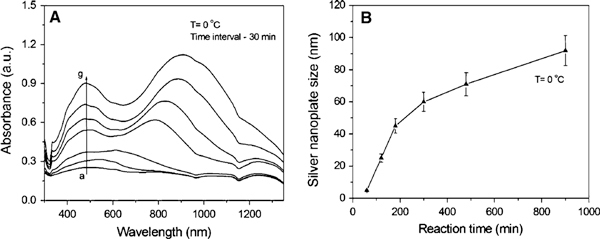
**a UV–vis spectra showing the growth of silver nanoparticles at ~0°C and b the size evolution of these particles with time**.

The corresponding UV–vis spectra recorded at ~0°C were shown in Figure 7a. It was found that the absorption intensity increases with time. The absorption band centred at 450 nm also increases significantly. The shift in the strongest absorption peak to longer wavelengths means the plate size increases, as shown in Figure 7b. Noting that the reaction took a long time (over tens of hours) to reach the end at such a low temperature. On the contrary, a high temperature over 60°C was not considered in this work because the higher the temperature the faster the reaction, hence the data are difficult to collect for TEM or UV–vis analysis. Generally speaking, the effect of temperature on the formation and growth of silver nanoparticles is significant under the reported conditions. Some components related to temperature still need to investigate in the future, such as the reducing ability of citric acid and ascorbic acid, the complex ability of citric acid or AOT surfactant molecules with silver ions. The thermodynamical parameters in this system, including Gibbs free energy (ΔG), enthalpy (ΔH), entropy (ΔS), and heat capacity (Cp), will be estimated quantitatively according to the TEM observations and UV–vis spectrum analysis. The results will be reported in our upcoming work.

## Conclusions

We have demonstrated the role of temperature played in the synthesis of silver nanoparticles in a synergetic reduction approach. The temperature can significantly affect the formation and growth, the shape, size, and size distribution of particles. This can be confirmed by TEM observations, UV–vis spectra measurements, and kinetics analysis. At the temperature range of 0–55°C, we found that (1) a low temperature (~0°C) could significantly slow down the formation and growth reaction, which usually takes tens of hours to complete the reducing reaction; (2) from 17 to 55°C, the reaction rate increases, and the particle size increases as well. There is a size jump at around 32°C for this reaction system, i.e., from ~90 to ~180 nm for the edge length of silver nanoplates and from 25 to 48 nm for the diameter of spheres; and (3) a high temperature (>60°C) was not investigated because at a higher temperature, for example, close to 100°C, citric acid will become more active in reducing silver ions [56,57]. This will lead to reaction too faster to collect data for TEM and/or UV–vis analysis.

In general, the heating or cooling of the reaction system will heavily affect the reaction capability of components in reduction, surfactant adsorption/desorption, and complexing stability, the formation and growth rate, and hence the shape, size, and size distributions. Much work should be performed in this area. The thermodynamical parameters, including Gibbs free energy (ΔG), enthalpy (ΔH), entropy (ΔS), and heat capacity (Cp), will be estimated quantitatively according to the TEM observations and UV–vis spectrum analysis and reported in our upcoming work. Nonetheless, the findings would be helpful to understand the effect of temperature on the formation and growth of silver nanoparticles. This approach could be extended into other systems associated with heating or cooling for shape/size and functional property control.
